# Synthesis, crystal structure and thermal properties of *catena*-poly[[[bis­(2,3-di­methyl­pyrazine-κ*N*^1^)cadmium(II)]-di-μ-chlorido] monohydrate]

**DOI:** 10.1107/S2056989026009941

**Published:** 2026-09-30

**Authors:** Christian Näther

**Affiliations:** a Institut für Anorganische Chemie, Universität Kiel, Max-Eyth.-Str. 2, 24118 Kiel, Germany; University of Aberdeen, United Kingdom

**Keywords:** coordination polymer, chain compound, synthesis, cadmium chloride, 2,3-di­methyl­pyrazine, crystal structure

## Abstract

The title compound, {[CdCl_2_(C_6_H_8_N_2_)_2_]·H_2_O}_*n*_ (C_6_H_8_N_2_ = 2,3-di­methyl­pyrazine) was prepared by the reaction of cadmium chloride with 2,3-di­methyl­pyrazine in water. The asymmetric unit consists of two crystallographically independent cadmium cations that are situated on a twofold rotation axis, two crystallographically independent chloride anions and 2,3-di­methyl­pyrazine ligands as well as one water mol­ecule that are all located in general positions. The cadmium cations are octa­hedrally coordinated by two terminally N-bonded 2,3-di­methyl­pyrazine ligands and four chloride anions and are linked by pairs of μ-1,1 bridging chloride anions into chains that propagate in the crystallographic *b*-axis direction. These chains are connected into layers *via* the water mol­ecule of crystallization by inter­chain O—H⋯N hydrogen bonding. Powder X-ray diffraction (PXRD) indicates that a pure phase has been obtained. Thermogravimetry (TG) coupled to differential thermal analysis (DTA) indicates that the water mol­ecules are removed in the first step and that the 2,3-di­methyl­pyrazine ligands are lost in the subsequent two steps, leading to a compound with the composition [CdCl_2_(C_6_H_8_N_2_)].

## Abstract 2

In the crystal structure of the title compound, the cadmium cations are linked by μ-1,1 bridging chloride anions into chains that are additionally connected by O—H⋯N hydrogen bonding *via* the water mol­ecules of crystallization into layers.

## 1. Chemical context

The crystal structures of transition-metal halide and pseudohalide coordination compounds are characterized by a large structural variability because the metal cations can be linked by bridging halide anions into metal-halide substructures of different dimensionality (Kromp & Sheldrick, 1999 ▸; Peng *et al.*, 2010 ▸; Li *et al.*, 2005 ▸; Näther *et al.*, 2002 ▸). This also leads to compounds of different stoichiometry in which the ratio between the metal halide and the coligand is different. In this context, thermal decomposition of coligand-rich compounds are useful to detect possible coligand-deficient compounds as inter­mediates because in several cases the coligands were removed in separate steps before a transformation into the pure metal halides is observed (Näther *et al.*, 2001 ▸; Näther & Jess, 2001 ▸).

In the course of our systematic work, we reported new cadmium halide coordination compounds with 2,3-di­methyl­pyrazine (C_6_H_8_N_2_) as ligand. With CdI_2_ we obtained two compounds with the compositions CdI_2_(C_6_H_8_N_2_)_2_ and CdI_2_(C_6_H_8_N_2_) (Näther, 2026*a* ▸). In both compounds the cadmium cations are tetra­hedrally coordinated, leading to discrete complexes in the 2,3-di­methyl­pyrazine-rich compound and which are linked into chains by the coligands in the 2,3-di­methyl­pyrazine-deficient compound. This is surprising because, in most cases, cadmium prefers an octa­hedral coordination and in this case one would expect the formation of CdI_2_ chains built up of CdI_4_N_2_ octa­hedra linked by common iodide edges. This is the case, for example, in CdI_2_(pyrazine), in which the cadmium cations are linked by pairs of μ-1,1 bridging halide anions into chains that are further connected into layers by the pyrazine ligands [CSD refcodes RINSOW, (Bailey & Pennington, 1997 ▸) and RINSOW01 (Pickardt & Staub, 1997 ▸)]. Starting from these results, we prepared the corresponding compounds with cadmium bromide and obtained a compound with the composition CdBr_2_(C_6_H_8_N_2_)(H_2_O)·C_6_H_8_N_2_·0.5H_2_O that consists of octa­hedrally coordinated cadmium cations that are linked into chains *via* pairs of bromide anions (Näther, 2026*b* ▸). In further work, CdBr_2_(C_6_H_8_N_2_) was structurally characterized. This compound exhibits the same ratio between the cadmium halide and the coligand as CdI_2_(C_6_H_8_N_2_) (Näther, 2026*c* ▸). In contrast to the latter compound, where the cadmium cations adopt a tetra­hedral coordination, for the bromide compound an octa­hedral coordination with a chain structure was observed. To complete these investigations, we became inter­ested in the corresponding compounds with CdCl_2_ and obtained crystals of the title compound, CdCl_2_(C_6_H_8_N_2_)_2_·H_2_O (**I**), which were characterized by single-crystal X-ray diffraction.
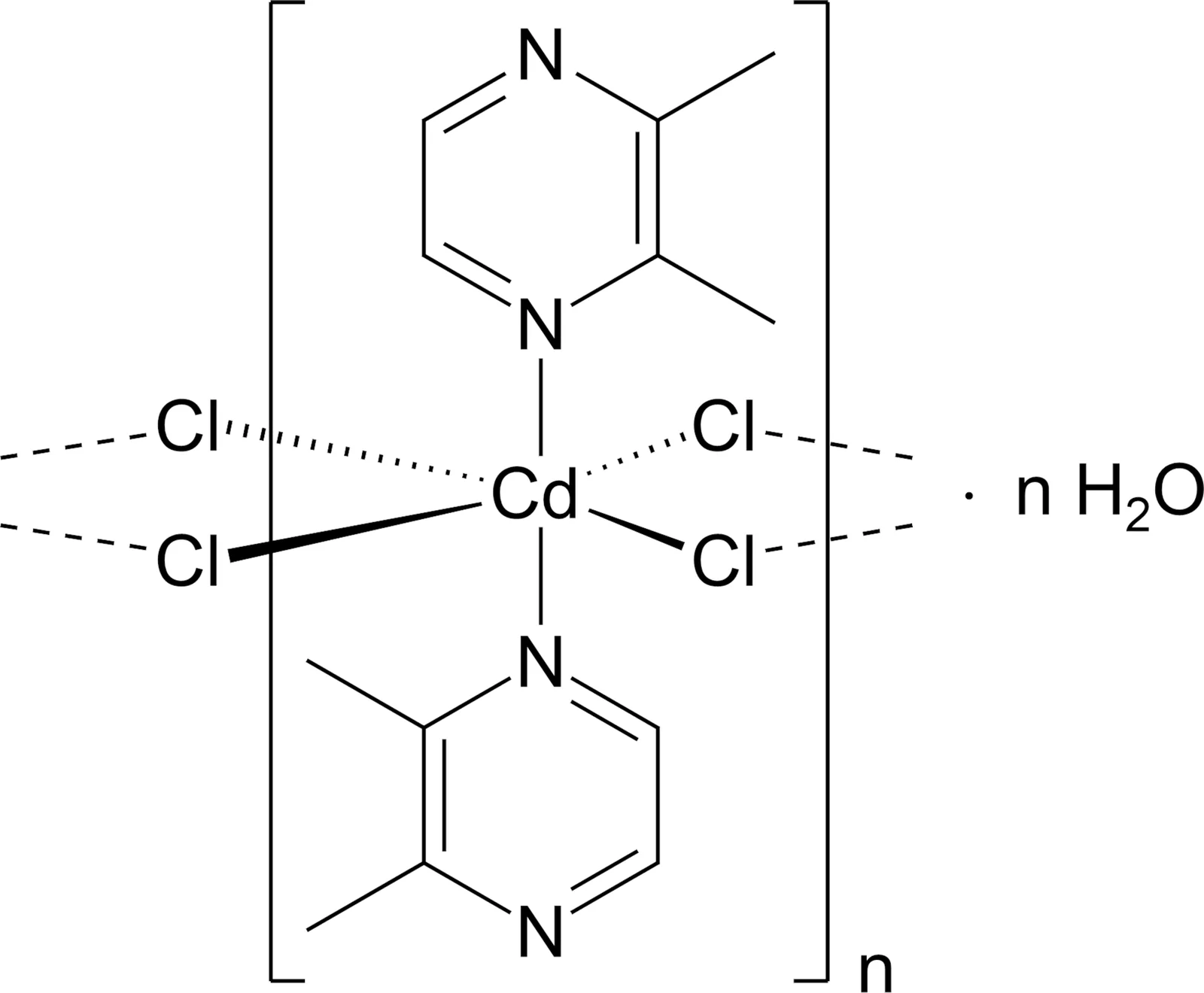


## 2. Structural commentary

The asymmetric unit of (**I**) is built up of two crystallographically independent cadmium cations that are both located on crystallographic twofold rotation axes, two crystallographically independent chloride anions and two distinct 2,3-di­methyl­pyrazine ligands as well as one water mol­ecule, all of them located in general positions. The cadmium cations are surrounded by four chloride anions and two terminally bonded 2,3-di­methyl­pyrazine ligands (Fig. 1 ▸). The bond angles show small deviations from the ideal octa­hedral values and there are no significant structural differences between the two crystallographically independent Cd centers (Table 1 ▸). The cadmium cations are linked by pairs of μ-1,1 bridging chloride anions into linear chains that propagate in the *b*-axis direction (Fig. 2 ▸).

This structure is therefore similar to that of CdBr_2_(C_6_H_8_N_2_), in which linear Cd*X* chains are also found (Näther, 2026*c* ▸). The major difference is that the 2,3-di­methyl­pyrazine ligands are only terminally bonded in the title compound, whereas they act as bridging ligands in the bromide compound and this is also reflected in the different ratio between Cd*X*_2_ and the 2,3-di­methyl­pyrazine coligand, which is 1:2 in the title compound and 1:1 in the bromide compound. Moreover, these findings also show that in cadmium halide coordination compounds with 2,3-di­methyl­pyrazine, chloride and bromide anions behave in a similar way with an octa­hedral Cd coordination and the presence of chains, whereas for the corresponding iodide compounds a tetra­hedral coordination is observed (Näther, 2026*a* ▸). At first glance one would assume that the reason why the iodide compound shows a tetra­hedral coordination might originate from steric repulsion between the larger iodide anions, which is lower in a tetra­hedral coordination, but this would not explain why in CdI_2_(pyrazine) an octa­hedral coordination is observed. One may therefore suggest that repulsion between the large iodide anions and the methyl group that is adjacent to the coordinating N atom is responsible for all these findings.

## 3. Supra­molecular features

In the extended structure of (**I**), the chains are linked *via* the water mol­ecules of crystallization into layers by O—H⋯N hydrogen bonds to the second N atom of the 2,3-di­methyl­pyrazine ligand that is not involved in the metal coordination (Fig. 3 ▸). These layers lie parallel to the *ab* plane (Fig. 3 ▸). The O—H⋯N bond angles are close to linear, indicating that this is a relatively strong inter­action (Table 2 ▸). There are also a number of C—H⋯Cl contacts, but most of them exhibit long distances and angles far from 180°, indicating only very weak inter­actions (Table 2 ▸). However, two of these contacts are relatively short with angles of 154 and 160°, indicating intra­chain hydrogen bonding (Table 2 ▸).

## 4. Additional characterization

The purity of the title compound was investigated by powder X-ray diffraction (PXRD) and comparison of the experimental PXRD pattern with that calculated from the results of the single crystal structure determination indicate that a pure phase has been obtained. However, some reflections are slightly shifted to higher Bragg angles in the calculated pattern, because of the different temperature and the pattern shows unusually strong preferred orientation (Fig. 4 ▸).

The thermal behavior of the title compound was investigated by thermogravimetry (TG) coupled to differential thermal analysis (DTA) to check if 2,3-di­methyl­pyrazine-deficient phases can be detected, as is frequently observed in such compounds. Upon heating three mass losses are observed, which are accompanied by endothermic events in the DTA curve (Fig. 5 ▸). The experimental mass loss in the first step of 4.23% is in very good agreement with that calculated for the removal of the water mol­ecules (4.31%) and the mass loss in the second step corresponds to the removal of 1.5 of the 2,3-di­methyl­pyrazine mol­ecules (Δ*m*_calc._ = 38.8%). Based on these results, one can expect that in the first step, a compound with the composition CdCl_2_(C_6_H_8_N_2_)_2_ is formed, which transforms into (CdCl_2_)_2_(C_6_H_8_N_2_) upon further heating.

The residues obtained after the first and second mass losses were investigated by PXRD. Comparison of the pattern of the residue with that of the pristine compound shows only very minor differences in the intensity and practically no shift in the reflection positions (Fig. S1). Two such experiments were performed with identical results. At first glance one would assume that this reaction might be topotactic, but at least some shift in peak positions would be expected. It is very unlikely that the title compound immediately decomposes on storage and that the anhydrate was measured because several TG-DTA experiments were performed using the same batch and in all of them the mass loss associated with removal of the water mol­ecules remain constant. On the other hand, we cannot exclude the possibility that the anhydrate is extremely hygroscopic and immediately transforms back to the title compound. The powder pattern of the residue obtained after the second mass loss is completely different from that of the title compound and shows that a powder of low crystallinity has formed (Fig. S2). The composition assumed for this residue seems to be a bit unusual and points to a more condensed CdCl_2_ substructure. In this context it is noted that, for the corresponding copper(I) chloride–2,3-di­methyl­pyrazine coordination compounds, a number of different compounds with varying ratios between CuCl and 2,3-di­methyl­pyrazine have been reported. These include (CuCl)_4_(C_6_H_8_N_2_)_9_ with a ratio of 4:9, CuCl(C_6_H_8_N_2_) with a ratio of 1:1, (CuCl)_3_(C_6_H_8_N_2_)_2_ with a ratio of 3:2 and (CuCl)_2_(C_6_H_8_N_2_) with a ratio of 2:1 (Jess & Näther, 2006 ▸).

## 5. Database survey

As mentioned above, several compounds with cadmium halides and 2,3-di­methyl­pyrazine have already been reported and this formed the basis for this work. This includes the iodide compounds CdI_2_(C_6_H_8_N_2_)_2_ and CdI_2_(C_6_H_8_N_2_) (Näther, 2026*a* ▸), as well as the bromide compounds CdBr_2_(C_6_H_8_N_2_)(H_2_O)·C_6_H_8_N_2_·0.5H_2_O and CdBr_2_(C_6_H_8_N_2_) (Näther, 2026*b* ▸,*c* ▸).

A search in the CSD (Version 5.43, 2025; Groom *et al.*, 2016 ▸) using CONQUEST (Bruno *et al.*, 2002 ▸) revealed that in several other compounds with pyrazine or pyridine and its derivatives, similar CdCl_2_ chains and an octa­hedral coordination is found. This is the case, for example, in CdCl_2_(pyridine)_2_, which shows the same chain structure as the title compound but contains no water of crystallization [CSD refcodes CDPYCL01 (Paulus, 1969 ▸), CDPYCL03 (Satoh *et al.*, 2001 ▸) and CDPYCL04 (Hu *et al.*, 2003 ▸)]. The same structure is also found in CdCl_2_(4-methyl­pyridine)_2_ (BASQUI; Satoh *et al.*, 2001 ▸), CdCl_2_(3-methyl­pyridine)_2_ [BASQOC (Satoh *et al.*, 2001 ▸), BASQOC01 (Hu *et al.*, 2003 ▸)]. CdCl_2_(pyrazine) [TISSUJ (Pickardt & Staub, 1996 ▸), TISSUJ01 (Bailey & Pennington, 1997 ▸)] is an additional example but in contrast to the pyridine compounds, the CdCl_2_ chains are linked into layers.

## 6. Synthesis, crystallization and experimental details

Cadmium chloride and 2,3-di­methyl­pyrazine were purchased from Sigma-Aldrich. To prepare (**I**), 1.00 mmol (183.3 mg) of CdCl_2_ and 1.00 mmol (108.2 mg) of 2,3-di­methyl­pyrazine were reacted in 2 ml of water for 3 d at room temperature, which led to the formation of crystals suitable for single-crystal structure analysis.

The PXRD measurements were performed with Cu *K*α_1_ radiation (λ = 1.540598 Å) using a Stoe Transmission Powder Diffraction System (STADI P) equipped with a MYTHEN 1K detector and a Johansson-type Ge(111) monochromator.

Thermogravimetry and differential thermal analysis (TG-DTA) measurements were performed in a dynamic air atmosphere in Al_2_O_3_ crucibles using a STA-PT 1000 thermobalance from Linseis. The instrument was calibrated using standard reference materials.

## 7. Refinement

Crystal data, data collection and structure refinement details are summarized in Table 3 ▸. The C—H hydrogen atoms were positioned with idealized geometry (methyl H atoms allowed to rotate but not to tip) and were refined isotropically with *U*_iso_(H) = 1.2*U*_eq_(C) (1.5 for methyl H atoms). The O-bound H atoms were located in difference maps and refined isotropically without restraints.

## Supplementary Material

Crystal structure: contains datablock(s) I. DOI: 10.1107/S2056989026009941/hb8257sup1.cif

Structure factors: contains datablock(s) I. DOI: 10.1107/S2056989026009941/hb8257Isup2.hkl

Figure S1. Experimental powder pattern of the residue obtained after water removal (top) and calculated pattern for the title compound (bottom). DOI: 10.1107/S2056989026009941/hb8257sup3.png

Figure S2. Experimental powder pattern of the residue obtained after the second mass loss in a TG measurement of the title compound. DOI: 10.1107/S2056989026009941/hb8257sup4.png

CCDC reference: 2590645

Additional supporting information:  crystallographic information; 3D view; checkCIF report

## Figures and Tables

**Figure 1:**
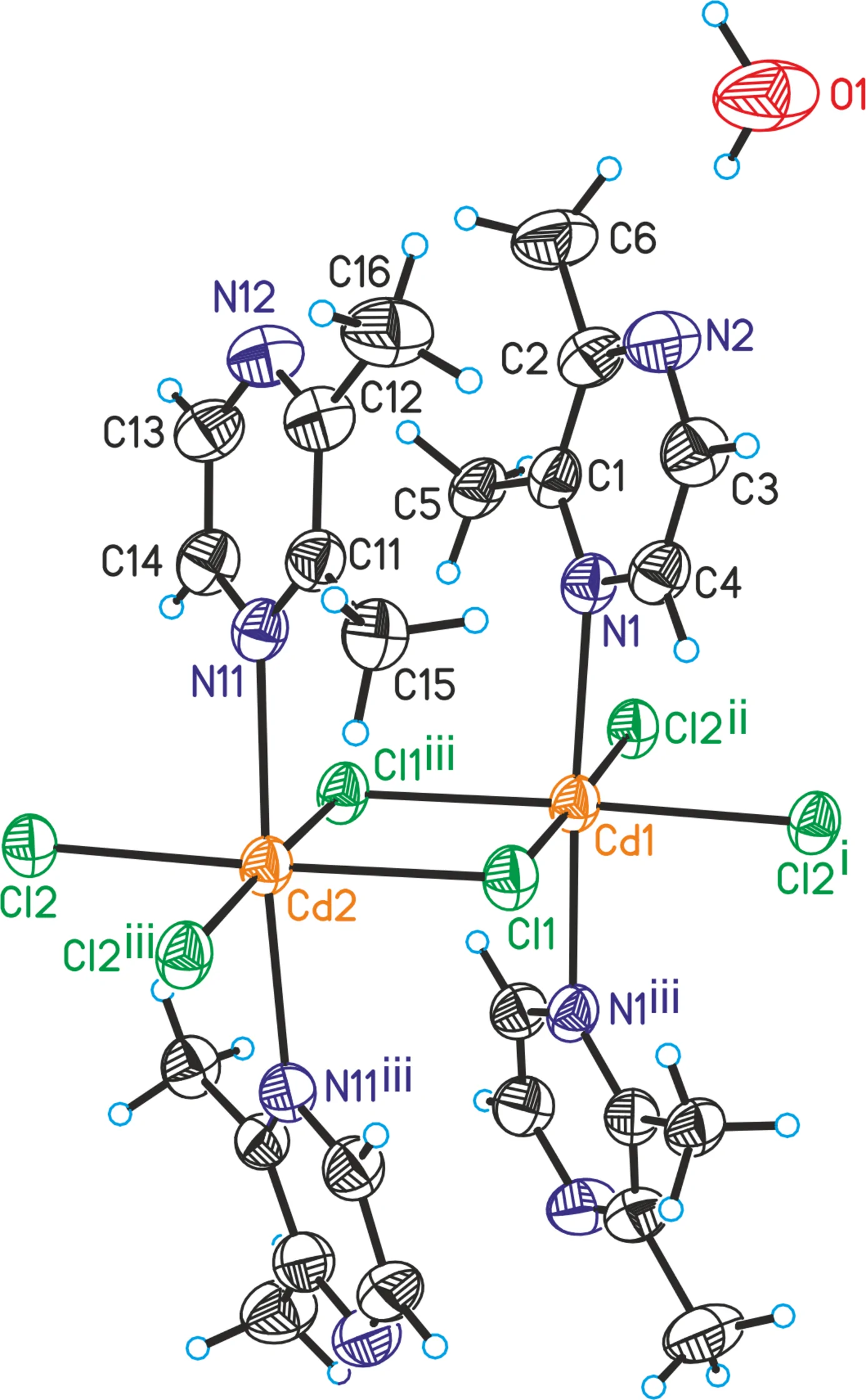
Crystal structure of (**I**) with labeling and displacement ellipsoids drawn at the 50% probability level. Symmetry codes: (i) −*x* + 1, *y* + 1, −*z* + 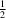; (ii) *x*, *y* + 1, *z*; (iii) −*x* + 1, *y*, −*z* + 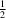.

**Figure 2:**
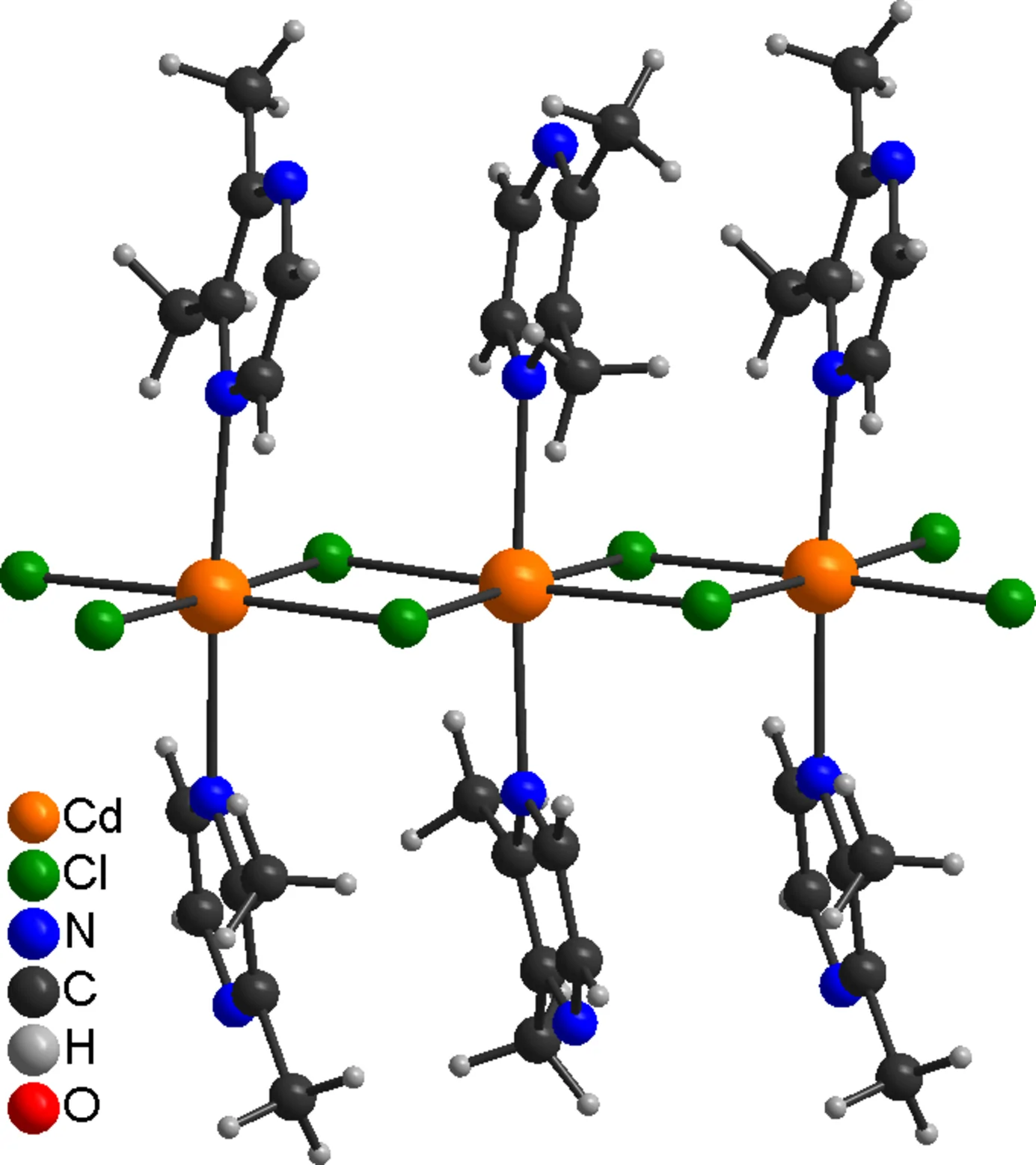
View of a part of a chain in the extended structure of (**I**).

**Figure 3:**
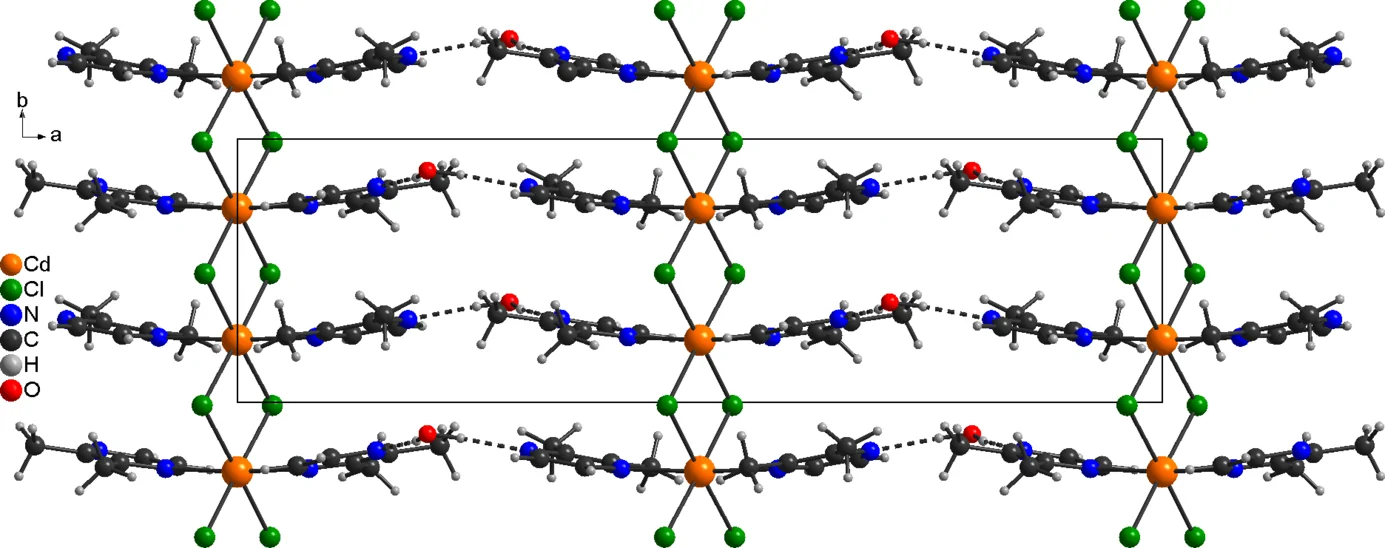
Crystal structure of (**I**) in a view along the crystallographic *a*-axis direction.

**Figure 4:**
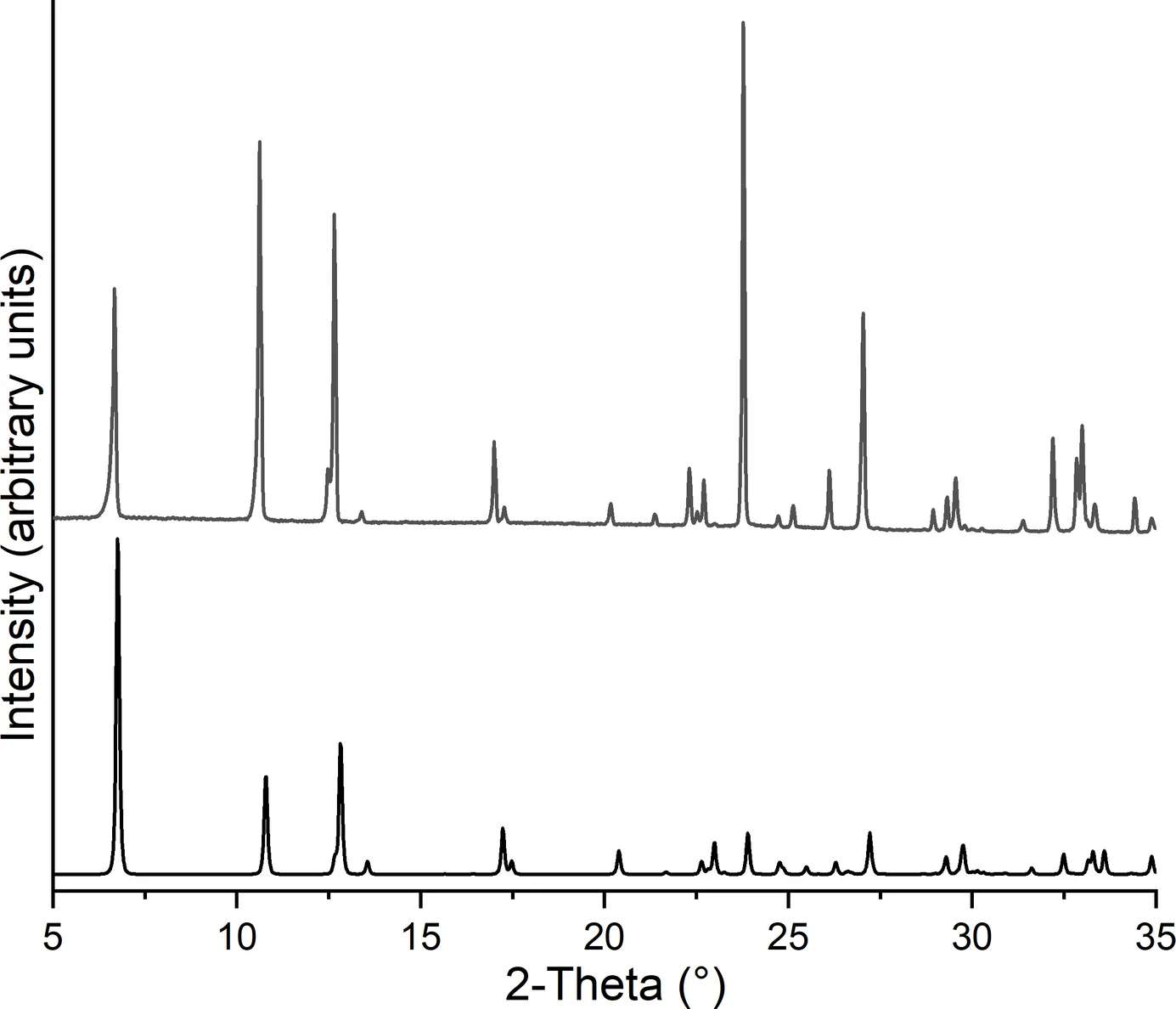
Experimental (top) and calculated powder patterns (bottom) for (**I**).

**Figure 5:**
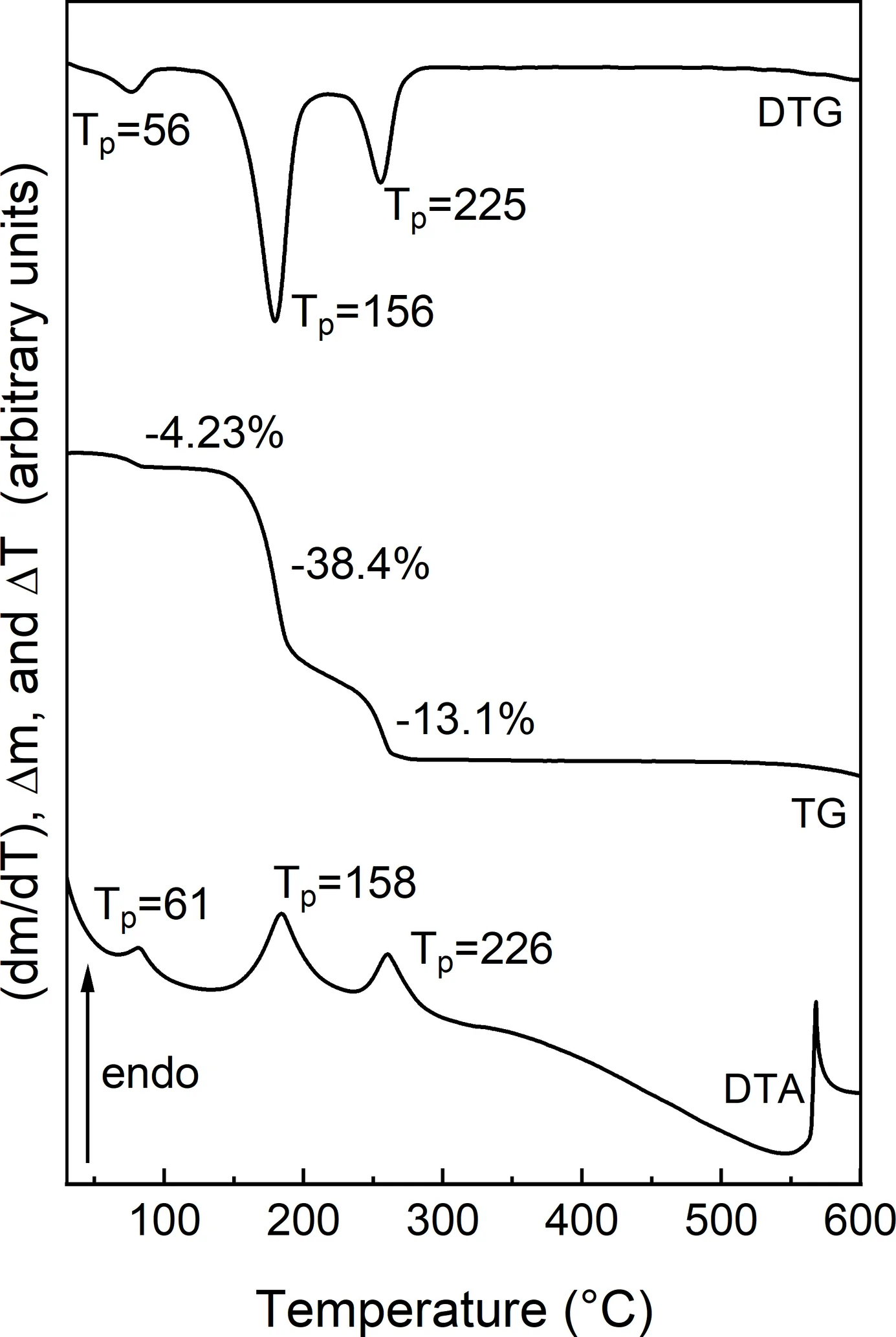
DTG, TG and DTA curves for (**I**).

**Table 1:** Selected geometric parameters (Å, °)

Cd1—N1	2.493 (3)	Cd2—N11	2.491 (3)
Cd1—Cl1	2.5773 (9)	Cd2—Cl2	2.5815 (9)
Cd1—Cl2^i^	2.5958 (9)	Cd2—Cl1	2.5930 (9)
Cd1—Cl2^ii^	2.5958 (9)		
			
N1—Cd1—N1^iii^	176.45 (11)	N11—Cd2—Cl2	88.11 (7)
N1—Cd1—Cl1^iii^	95.65 (7)	N11^iii^—Cd2—Cl2	94.93 (7)
N1—Cd1—Cl1	86.91 (7)	Cl2—Cd2—Cl2^iii^	88.64 (4)
Cl1^iii^—Cd1—Cl1	88.14 (4)	N11—Cd2—Cl1	89.88 (7)
N1—Cd1—Cl2^i^	86.33 (7)	N11^iii^—Cd2—Cl1	87.06 (7)
N1^iii^—Cd1—Cl2^i^	91.11 (7)	Cl2—Cd2—Cl1	177.93 (2)
Cl1^iii^—Cd1—Cl2^i^	178.02 (2)	Cl2^iii^—Cd2—Cl1	91.98 (3)
Cl1—Cd1—Cl2^i^	91.95 (3)	Cl2^iii^—Cd2—Cl1^iii^	177.93 (2)
Cl1—Cd1—Cl2^ii^	178.02 (2)	Cl1—Cd2—Cl1^iii^	87.47 (4)
Cl2^i^—Cd1—Cl2^ii^	88.03 (4)	Cd1—Cl1—Cd2	92.20 (3)
N11—Cd2—N11^iii^	175.76 (11)	Cd2—Cl2—Cd1^iv^	91.66 (3)

Symmetry codes: (i) 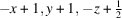; (ii) 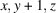; (iii) 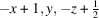; (iv) 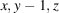.

**Table 2:** Hydrogen-bond geometry (Å, °)

*D*—H⋯*A*	*D*—H	H⋯*A*	*D*⋯*A*	*D*—H⋯*A*
O1—H1*A*⋯N12^v^	1.00 (7)	2.00 (7)	2.965 (4)	164 (5)
O1—H1*B*⋯N2	0.94 (6)	2.02 (7)	2.939 (5)	165 (5)
C3—H3⋯O1^vi^	0.95	2.61	3.282 (5)	128
C4—H4⋯Cl1	0.95	2.93	3.490 (4)	119
C4—H4⋯Cl2^i^	0.95	2.97	3.420 (4)	110
C5—H5*B*⋯Cl1^vii^	0.98	2.88	3.747 (3)	147
C5—H5*C*⋯Cl1^iii^	0.98	2.71	3.650 (4)	160
C5—H5*C*⋯Cl2^ii^	0.98	2.97	3.451 (4)	112
C13—H13⋯Cl1^vii^	0.95	2.98	3.786 (4)	143
C14—H14⋯Cl1^iii^	0.95	2.95	3.419 (4)	112
C14—H14⋯Cl2	0.95	2.94	3.510 (4)	120
C15—H15*A*⋯Cl1	0.98	2.90	3.470 (4)	118
C15—H15*A*⋯Cl2^iii^	0.98	2.76	3.670 (4)	154

Symmetry codes: (i) 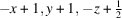; (ii) 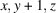; (iii) 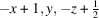; (v) 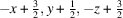; (vi) 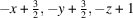; (vii) 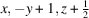.

**Table 3:** Experimental details

Crystal data	
Chemical formula	[CdCl_2_(C_6_H_8_N_2_)_2_]·H_2_O
*M*_r_	417.60
Crystal system, space group	Monoclinic, *C*2/*c*
Temperature (K)	170
*a*, *b*, *c* (Å)	30.6316 (15), 7.4391 (3), 16.3857 (8)
β (°)	121.538 (3)
*V* (Å^3^)	3182.3 (3)
*Z*	8
Radiation type	Mo *K*α
μ (mm^−1^)	1.71
Crystal size (mm)	0.14 × 0.12 × 0.09

Data collection	
Diffractometer	*IPDS2*
Absorption correction	Numerical (*X-RED* and *X-SHAPE*; Stoe, 2002 ▸)
*T*_min_, *T*_max_	0.719, 0.812
No. of measured, independent and observed [*I* > 2σ(*I*)] reflections	24424, 3836, 2792
*R*_int_	0.111
(sin θ/λ)_max_ (Å^−1^)	0.661

Refinement	
*R*[*F*^2^ > 2σ(*F*^2^)], *wR*(*F*^2^), *S*	0.047, 0.143, 1.03
No. of reflections	3836
No. of parameters	195
H-atom treatment	H atoms treated by a mixture of independent and constrained refinement
Δρ_max_, Δρ_min_ (e Å^−3^)	1.03, −1.76

Computer programs: *X-AREA* (Stoe, 2002 ▸), *SHELXT2014/4* (Sheldrick, 2015*a* ▸), *SHELXL2016/6* (Sheldrick, 2015*b* ▸), *DIAMOND* (Brandenburg, 1999 ▸), *XP* in *SHELXTL-PC* (Sheldrick, 2008 ▸) and *publCIF* (Westrip, 2010 ▸).

## supplementary crystallographic information

### catena-Poly[[[bis(2,3-dimethylpyrazine-κN1)cadmium(II)]-di-µ-chlorido] monohydrate] Crystal data

**Table:**

[CdCl_2_(C_6_H_8_N_2_)_2_]·H_2_O	*F*(000) = 1664
*M**_r_* = 417.60	*D*_x_ = 1.743 Mg m^−^^3^
Monoclinic, *C*2/*c*	Mo *K*α radiation, λ = 0.71073 Å
*a* = 30.6316 (15) Å	Cell parameters from 22970 reflections
*b* = 7.4391 (3) Å	θ = 1.5–28.9°
*c* = 16.3857 (8) Å	µ = 1.71 mm^−^^1^
β = 121.538 (3)°	*T* = 170 K
*V* = 3182.3 (3) Å^3^	Block, colorless
*Z* = 8	0.14 × 0.12 × 0.09 mm

### catena-Poly[[[bis(2,3-dimethylpyrazine-κN1)cadmium(II)]-di-µ-chlorido] monohydrate] Data collection

**Table:**

IPDS-2 diffractometer	2792 reflections with *I* > 2σ(*I*)
ω scans	*R*_int_ = 0.111
Absorption correction: numerical (X-Red and X-Shape; Stoe, 2002)	θ_max_ = 28.0°, θ_min_ = 1.6°
*T*_min_ = 0.719, *T*_max_ = 0.812	*h* = −40→40
24424 measured reflections	*k* = −8→9
3836 independent reflections	*l* = −21→21

### catena-Poly[[[bis(2,3-dimethylpyrazine-κN1)cadmium(II)]-di-µ-chlorido] monohydrate] Refinement

**Table:**

Refinement on *F*^2^	Hydrogen site location: mixed
Least-squares matrix: full	H atoms treated by a mixture of independent and constrained refinement
*R*[*F*^2^ > 2σ(*F*^2^)] = 0.047	*w* = 1/[σ^2^(*F*_o_^2^) + (0.0824*P*)^2^] where *P* = (*F*_o_^2^ + 2*F*_c_^2^)/3
*wR*(*F*^2^) = 0.143	(Δ/σ)_max_ = 0.001
*S* = 1.03	Δρ_max_ = 1.03 e Å^−^^3^
3836 reflections	Δρ_min_ = −1.76 e Å^−^^3^
195 parameters	Extinction correction: *SHELXL2016/6* (Sheldrick, 2015b), Fc^*^=kFc[1+0.001xFc^2^λ^3^/sin(2θ)]^-1/4^
0 restraints	Extinction coefficient: 0.00097 (15)

### catena-Poly[[[bis(2,3-dimethylpyrazine-κN1)cadmium(II)]-di-µ-chlorido] monohydrate] Special details

**Table:**

Geometry. All esds (except the esd in the dihedral angle between two l.s. planes) are estimated using the full covariance matrix. The cell esds are taken into account individually in the estimation of esds in distances, angles and torsion angles; correlations between esds in cell parameters are only used when they are defined by crystal symmetry. An approximate (isotropic) treatment of cell esds is used for estimating esds involving l.s. planes.

### catena-Poly[[[bis(2,3-dimethylpyrazine-κN1)cadmium(II)]-di-µ-chlorido] monohydrate] Fractional atomic coordinates and isotropic or equivalent isotropic displacement parameters (Å^2^)

**Table:**

	*x*	*y*	*z*	*U*_iso_*/*U*_eq_	
Cd1	0.500000	0.73731 (5)	0.250000	0.03384 (15)	
Cd2	0.500000	0.23652 (5)	0.250000	0.03428 (15)	
Cl1	0.53794 (4)	0.48838 (10)	0.19592 (7)	0.0374 (2)	
Cl2	0.46474 (4)	−0.01175 (10)	0.31017 (7)	0.0374 (2)	
N1	0.58483 (13)	0.7477 (3)	0.4026 (2)	0.0375 (7)	
C1	0.59557 (14)	0.7695 (4)	0.4922 (2)	0.0373 (7)	
C2	0.64592 (14)	0.8044 (6)	0.5672 (2)	0.0467 (8)	
N2	0.68450 (12)	0.8149 (5)	0.5519 (2)	0.0536 (8)	
C3	0.67300 (14)	0.7877 (5)	0.4618 (3)	0.0469 (8)	
H3	0.699816	0.790312	0.448839	0.056*	
C4	0.62419 (15)	0.7566 (4)	0.3882 (3)	0.0413 (8)	
H4	0.617810	0.740743	0.325322	0.050*	
C5	0.55300 (15)	0.7540 (4)	0.5117 (3)	0.0417 (9)	
H5A	0.545233	0.873110	0.526423	0.063*	
H5B	0.563519	0.673542	0.566231	0.063*	
H5C	0.522444	0.705258	0.455015	0.063*	
C6	0.65882 (17)	0.8313 (7)	0.6686 (3)	0.0669 (12)	
H6A	0.660295	0.714319	0.697424	0.100*	
H6B	0.632439	0.905913	0.668492	0.100*	
H6C	0.692140	0.891136	0.705752	0.100*	
N11	0.57755 (12)	0.2489 (3)	0.4142 (2)	0.0368 (7)	
C11	0.62713 (14)	0.2707 (4)	0.4435 (2)	0.0384 (8)	
C12	0.66333 (13)	0.3077 (6)	0.5407 (2)	0.0449 (8)	
N12	0.65036 (12)	0.3168 (5)	0.6062 (2)	0.0503 (7)	
C13	0.60089 (15)	0.2888 (5)	0.5756 (2)	0.0470 (8)	
H13	0.590506	0.289731	0.620968	0.056*	
C14	0.56478 (16)	0.2589 (5)	0.4813 (3)	0.0431 (9)	
H14	0.529946	0.244861	0.462766	0.052*	
C15	0.64336 (14)	0.2545 (4)	0.3723 (3)	0.0423 (8)	
H15A	0.614010	0.217636	0.310243	0.064*	
H15B	0.670571	0.164260	0.394365	0.064*	
H15C	0.656145	0.370793	0.365481	0.064*	
C16	0.71882 (14)	0.3366 (7)	0.5745 (3)	0.0619 (11)	
H16A	0.734999	0.408587	0.633216	0.093*	
H16B	0.721521	0.399934	0.524842	0.093*	
H16C	0.736137	0.220025	0.587352	0.093*	
O1	0.79443 (13)	0.8800 (6)	0.6842 (2)	0.0899 (13)	
H1A	0.806 (2)	0.853 (9)	0.752 (5)	0.12 (2)*	
H1B	0.759 (2)	0.864 (8)	0.651 (4)	0.108 (19)*	

### catena-Poly[[[bis(2,3-dimethylpyrazine-κN1)cadmium(II)]-di-µ-chlorido] monohydrate] Atomic displacement parameters (Å^2^)

**Table:**

	*U*^11^	*U*^22^	*U*^33^	*U*^12^	*U*^13^	*U*^23^
Cd1	0.0430 (2)	0.0296 (2)	0.0362 (2)	0.000	0.02573 (18)	0.000
Cd2	0.0436 (3)	0.0299 (2)	0.0362 (2)	0.000	0.02563 (19)	0.000
Cl1	0.0490 (5)	0.0325 (5)	0.0419 (5)	−0.0010 (3)	0.0315 (4)	−0.0015 (3)
Cl2	0.0484 (5)	0.0323 (5)	0.0417 (5)	−0.0006 (3)	0.0307 (4)	−0.0013 (3)
N1	0.0498 (18)	0.0335 (17)	0.0373 (16)	0.0001 (11)	0.0284 (14)	−0.0009 (10)
C1	0.0463 (19)	0.0342 (19)	0.0381 (17)	0.0016 (13)	0.0267 (16)	0.0032 (12)
C2	0.053 (2)	0.053 (2)	0.0357 (16)	0.0010 (17)	0.0245 (15)	0.0019 (16)
N2	0.0452 (16)	0.072 (2)	0.0395 (15)	−0.0004 (17)	0.0194 (13)	0.0003 (16)
C3	0.0452 (19)	0.054 (2)	0.0474 (19)	0.0022 (16)	0.0285 (17)	0.0023 (16)
C4	0.049 (2)	0.044 (2)	0.0389 (18)	0.0022 (13)	0.0281 (17)	0.0020 (13)
C5	0.055 (2)	0.046 (2)	0.0363 (17)	0.0021 (14)	0.0320 (17)	0.0028 (12)
C6	0.066 (3)	0.094 (4)	0.0358 (18)	−0.001 (2)	0.0232 (18)	−0.005 (2)
N11	0.0425 (16)	0.0355 (18)	0.0387 (16)	0.0018 (10)	0.0257 (14)	0.0027 (10)
C11	0.0457 (19)	0.0344 (19)	0.0364 (17)	0.0019 (13)	0.0225 (15)	0.0010 (12)
C12	0.0425 (18)	0.050 (2)	0.0426 (17)	0.0039 (16)	0.0223 (15)	0.0005 (16)
N12	0.0491 (16)	0.062 (2)	0.0381 (15)	0.0037 (15)	0.0213 (13)	−0.0010 (15)
C13	0.054 (2)	0.055 (2)	0.0370 (17)	0.0032 (17)	0.0274 (16)	−0.0001 (15)
C14	0.048 (2)	0.045 (2)	0.044 (2)	0.0037 (14)	0.0300 (18)	0.0020 (13)
C15	0.0422 (19)	0.045 (2)	0.048 (2)	0.0013 (13)	0.0294 (18)	0.0007 (13)
C16	0.045 (2)	0.086 (3)	0.049 (2)	0.000 (2)	0.0210 (17)	−0.005 (2)
O1	0.0568 (19)	0.168 (4)	0.0464 (16)	−0.017 (2)	0.0282 (15)	0.002 (2)

### catena-Poly[[[bis(2,3-dimethylpyrazine-κN1)cadmium(II)]-di-µ-chlorido] monohydrate] Geometric parameters (Å, º)

**Table:**

Cd1—N1	2.493 (3)	C5—H5B	0.9800
Cd1—N1^i^	2.493 (3)	C5—H5C	0.9800
Cd1—Cl1^i^	2.5773 (9)	C6—H6A	0.9800
Cd1—Cl1	2.5773 (9)	C6—H6B	0.9800
Cd1—Cl2^ii^	2.5958 (9)	C6—H6C	0.9800
Cd1—Cl2^iii^	2.5958 (9)	N11—C11	1.342 (5)
Cd2—N11	2.491 (3)	N11—C14	1.348 (5)
Cd2—N11^i^	2.491 (3)	C11—C12	1.412 (5)
Cd2—Cl2	2.5815 (9)	C11—C15	1.494 (5)
Cd2—Cl2^i^	2.5815 (9)	C12—N12	1.328 (4)
Cd2—Cl1	2.5930 (9)	C12—C16	1.503 (5)
Cd2—Cl1^i^	2.5930 (9)	N12—C13	1.340 (5)
N1—C1	1.337 (4)	C13—C14	1.367 (5)
N1—C4	1.346 (5)	C13—H13	0.9500
C1—C2	1.404 (5)	C14—H14	0.9500
C1—C5	1.500 (5)	C15—H15A	0.9800
C2—N2	1.332 (5)	C15—H15B	0.9800
C2—C6	1.506 (4)	C15—H15C	0.9800
N2—C3	1.342 (5)	C16—H16A	0.9800
C3—C4	1.363 (5)	C16—H16B	0.9800
C3—H3	0.9500	C16—H16C	0.9800
C4—H4	0.9500	O1—H1A	1.00 (7)
C5—H5A	0.9800	O1—H1B	0.94 (6)
			
N1—Cd1—N1^i^	176.45 (11)	N1—C4—C3	121.5 (3)
N1—Cd1—Cl1^i^	95.65 (7)	N1—C4—H4	119.3
N1^i^—Cd1—Cl1^i^	86.91 (7)	C3—C4—H4	119.3
N1—Cd1—Cl1	86.91 (7)	C1—C5—H5A	109.5
N1^i^—Cd1—Cl1	95.65 (7)	C1—C5—H5B	109.5
Cl1^i^—Cd1—Cl1	88.14 (4)	H5A—C5—H5B	109.5
N1—Cd1—Cl2^ii^	86.33 (7)	C1—C5—H5C	109.5
N1^i^—Cd1—Cl2^ii^	91.11 (7)	H5A—C5—H5C	109.5
Cl1^i^—Cd1—Cl2^ii^	178.02 (2)	H5B—C5—H5C	109.5
Cl1—Cd1—Cl2^ii^	91.95 (3)	C2—C6—H6A	109.5
N1—Cd1—Cl2^iii^	91.11 (7)	C2—C6—H6B	109.5
N1^i^—Cd1—Cl2^iii^	86.33 (7)	H6A—C6—H6B	109.5
Cl1^i^—Cd1—Cl2^iii^	91.95 (3)	C2—C6—H6C	109.5
Cl1—Cd1—Cl2^iii^	178.02 (2)	H6A—C6—H6C	109.5
Cl2^ii^—Cd1—Cl2^iii^	88.03 (4)	H6B—C6—H6C	109.5
N11—Cd2—N11^i^	175.76 (11)	C11—N11—C14	117.3 (3)
N11—Cd2—Cl2	88.11 (7)	C11—N11—Cd2	130.7 (2)
N11^i^—Cd2—Cl2	94.93 (7)	C14—N11—Cd2	111.3 (3)
N11—Cd2—Cl2^i^	94.93 (7)	N11—C11—C12	120.1 (3)
N11^i^—Cd2—Cl2^i^	88.11 (7)	N11—C11—C15	119.2 (3)
Cl2—Cd2—Cl2^i^	88.64 (4)	C12—C11—C15	120.7 (3)
N11—Cd2—Cl1	89.88 (7)	N12—C12—C11	122.1 (3)
N11^i^—Cd2—Cl1	87.06 (7)	N12—C12—C16	117.0 (3)
Cl2—Cd2—Cl1	177.93 (2)	C11—C12—C16	120.9 (3)
Cl2^i^—Cd2—Cl1	91.98 (3)	C12—N12—C13	116.5 (3)
N11—Cd2—Cl1^i^	87.06 (7)	N12—C13—C14	122.5 (3)
N11^i^—Cd2—Cl1^i^	89.87 (7)	N12—C13—H13	118.8
Cl2—Cd2—Cl1^i^	91.98 (3)	C14—C13—H13	118.8
Cl2^i^—Cd2—Cl1^i^	177.93 (2)	N11—C14—C13	121.4 (4)
Cl1—Cd2—Cl1^i^	87.47 (4)	N11—C14—H14	119.3
Cd1—Cl1—Cd2	92.20 (3)	C13—C14—H14	119.3
Cd2—Cl2—Cd1^iv^	91.66 (3)	C11—C15—H15A	109.5
C1—N1—C4	117.3 (3)	C11—C15—H15B	109.5
C1—N1—Cd1	129.4 (3)	H15A—C15—H15B	109.5
C4—N1—Cd1	112.7 (2)	C11—C15—H15C	109.5
N1—C1—C2	120.6 (3)	H15A—C15—H15C	109.5
N1—C1—C5	118.8 (3)	H15B—C15—H15C	109.5
C2—C1—C5	120.5 (3)	C12—C16—H16A	109.5
N2—C2—C1	121.5 (3)	C12—C16—H16B	109.5
N2—C2—C6	116.9 (3)	H16A—C16—H16B	109.5
C1—C2—C6	121.6 (3)	C12—C16—H16C	109.5
C2—N2—C3	116.7 (3)	H16A—C16—H16C	109.5
N2—C3—C4	122.3 (3)	H16B—C16—H16C	109.5
N2—C3—H3	118.8	H1A—O1—H1B	104 (4)
C4—C3—H3	118.8		
			
C4—N1—C1—C2	1.4 (5)	C14—N11—C11—C12	−1.8 (5)
Cd1—N1—C1—C2	−168.3 (3)	Cd2—N11—C11—C12	167.8 (2)
C4—N1—C1—C5	−177.9 (3)	C14—N11—C11—C15	177.9 (3)
Cd1—N1—C1—C5	12.4 (4)	Cd2—N11—C11—C15	−12.5 (4)
N1—C1—C2—N2	−0.7 (6)	N11—C11—C12—N12	2.0 (6)
C5—C1—C2—N2	178.5 (4)	C15—C11—C12—N12	−177.6 (4)
N1—C1—C2—C6	179.4 (4)	N11—C11—C12—C16	−178.6 (4)
C5—C1—C2—C6	−1.3 (6)	C15—C11—C12—C16	1.7 (6)
C1—C2—N2—C3	−1.0 (6)	C11—C12—N12—C13	0.1 (6)
C6—C2—N2—C3	178.9 (4)	C16—C12—N12—C13	−179.2 (4)
C2—N2—C3—C4	2.1 (6)	C12—N12—C13—C14	−2.4 (6)
C1—N1—C4—C3	−0.4 (5)	C11—N11—C14—C13	−0.4 (5)
Cd1—N1—C4—C3	171.0 (3)	Cd2—N11—C14—C13	−172.0 (3)
N2—C3—C4—N1	−1.4 (6)	N12—C13—C14—N11	2.7 (6)

Symmetry codes: (i) −*x*+1, *y*, −*z*+1/2; (ii) −*x*+1, *y*+1, −*z*+1/2; (iii) *x*, *y*+1, *z*; (iv) *x*, *y*−1, *z*.

### catena-Poly[[[bis(2,3-dimethylpyrazine-κN1)cadmium(II)]-di-µ-chlorido] monohydrate] Hydrogen-bond geometry (Å, º)

**Table:**

*D*—H···*A*	*D*—H	H···*A*	*D*···*A*	*D*—H···*A*
O1—H1*A*···N12^v^	1.00 (7)	2.00 (7)	2.965 (4)	164 (5)
O1—H1*B*···N2	0.94 (6)	2.02 (7)	2.939 (5)	165 (5)
C3—H3···O1^vi^	0.95	2.61	3.282 (5)	128
C4—H4···Cl1	0.95	2.93	3.490 (4)	119
C4—H4···Cl2^ii^	0.95	2.97	3.420 (4)	110
C5—H5*B*···Cl1^vii^	0.98	2.88	3.747 (3)	147
C5—H5*C*···Cl1^i^	0.98	2.71	3.650 (4)	160
C5—H5*C*···Cl2^iii^	0.98	2.97	3.451 (4)	112
C13—H13···Cl1^vii^	0.95	2.98	3.786 (4)	143
C14—H14···Cl1^i^	0.95	2.95	3.419 (4)	112
C14—H14···Cl2	0.95	2.94	3.510 (4)	120
C15—H15*A*···Cl1	0.98	2.90	3.470 (4)	118
C15—H15*A*···Cl2^i^	0.98	2.76	3.670 (4)	154

Symmetry codes: (i) −*x*+1, *y*, −*z*+1/2; (ii) −*x*+1, *y*+1, −*z*+1/2; (iii) *x*, *y*+1, *z*; (v) −*x*+3/2, *y*+1/2, −*z*+3/2; (vi) −*x*+3/2, −*y*+3/2, −*z*+1; (vii) *x*, −*y*+1, *z*+1/2.
